# Discovery of novel *IDH1*-R132C inhibitors through structure-based virtual screening

**DOI:** 10.3389/fphar.2022.982375

**Published:** 2022-09-07

**Authors:** Chujiao Hu, Zhirui Zeng, Dan Ma, Zhixin Yin, Shanshan Zhao, Tengxiang Chen, Lei Tang, Shi Zuo

**Affiliations:** ^1^ Department of Hepatobiliary Surgery, The Affiliated Hospital of Guizhou Medical University, Guiyang, China; ^2^ State Key Laboratory of Functions and Applications of Medicinal Plants, Guizhou Medical University, Guiyang, China; ^3^ Guizhou Provincial Engineering Technology Research Center for Chemical Drug R and D, Guiyang, China; ^4^ Transformation Engineering Research Center of Chronic Disease Diagnosis and Treatment, Department of Physiology, School of Basic Medical Sciences, Guizhou Medical University, Guiyang, China; ^5^ Precision Medicine Research Institute of Guizhou, The Affiliated Hospital of Guizhou Medical University, Guiyang, China; ^6^ Department of Hematology, The Affiliated Hospital of Guizhou Medical University, Guiyang, China; ^7^ College of Pharmacy, Guizhou Medical University, Guiyang, China

**Keywords:** IDH1-R132C inhibitor, virtual screening, molecular docking, molecular dynamics simulation, tricarboxylic acid cycle (TCA cycle)

## Abstract

Isocitrate dehydrogenase (*IDH*) belongs to a family of enzymes involved in glycometabolism. It is found in many living organisms and is one of the most mutated metabolic enzymes. In the current study, we identified novel *IDH*1-R132C inhibitors using docking-based virtual screening and cellular inhibition assays. A total of 100 molecules with high docking scores were obtained from docking-based virtual screening. The cellular inhibition assay demonstrated five compounds at a concentration of 10 μM could inhibit cancer cells harboring the *IDH*1-R132C mutation proliferation by > 50%. The compound (T001-0657) showed the most potent effect against cancer cells harboring the *IDH*1-R132C mutation with a half-maximal inhibitory concentration (IC_50_) value of 1.311 μM. It also showed a cytotoxic effect against cancer cells with wild-type *IDH*1 and normal cells with IC_50_ values of 49.041 μM and >50 μM, respectively. Molecular dynamics simulations were performed to investigate the stability of the kinase structure binding of allosteric inhibitor compound A and the identified compound T001-0657 binds to *IDH*1-R132C. Root-mean-square deviation, root-mean-square fluctuation, and binding free energy calculations showed that both compounds bind tightly to *IDH*1-R132C. In conclusion, the compound identified in this study had high selectivity for cancer cells harboring *IDH*1-R132C mutation and could be considered a promising hit compound for further development of *IDH*1-R132C inhibitors.

## Introduction

Abnormalities in metabolism are one of the top ten characteristics of malignant tumors. Some metabolic enzymes act as oncogenes, promote tumorigenesis, and serve as attractive therapeutic targets (Krogan et al., 2015). Therefore, exploring small molecules which can inhibit the activity of these oncogenic metabolic enzymes may contribute to cancer therapy (Santos et al., 2017).

Isocitrate dehydrogenase (*IDH*) belongs to a family of enzymes involved in the tricarboxylic acid cycle (TCA). It is found in various living organisms and is one of the most frequently mutated metabolic enzymes of the TCA cycle across human cancers. The *IDH* mutations are somatic, heterozygous, and typically affect specific arginine residues (*IDH*1 R132 and *IDH*2 R140 or R172). Both catalyzes the oxidative decarboxylation of isocitrate to alpha-ketoglutarate (α-KG). However, *IDH*1 and *IDH*2 have distinctive physiological roles and cellular localization. *IDH*1 is localized in the cytoplasm, while the localization of *IDH*2 is mitochondrial. In most cases, the *IDH*1 and *IDH*2 mutations are mutually exclusive (Reitman and Yan 2010; Papaemmanuil et al., 2016). They are homodimers that utilize reduced nicotinamide adenine dinucleotide phosphate (NADP+) as a coenzyme to accept electrons. The *IDH* dimer consists of two asymmetric monomers. Each dimer contains three structural domains, of which one of them is a large structural domain (Yang et al., 2010). The wild-type *IDH* catalyzes the formation of α-KG, carbon dioxide (CO_2_), and Nicotinamide adenine dinucleotide phosphate (NADPH) from cytosolic isocitrate *via* NADP + -dependent oxidative decarboxylation. Thus, *IDH* significantly impacts the biosynthesis of metabolites and the production of cellular NADPH at the center of the TCA (Dang et al., 2016; Mondesir et al., 2016). Over 90% of mutations occur at the R132 residue of *IDH*1, of which mutations to histidine (R132H) are the most common (Parsons et al., 2008). Less frequently occurring mutations include R132C, R132G, R132L, and R132S. Mutations in *IDH* were first reported in colon cancers; however, several studies have now reported mutations in *IDH*1 and *IDH*2 in several cancers, including gliomas (Yan et al., 2009; Ward et al., 2010), intrahepatic acute myeloid leukemia (Abbas et al., 2010; Amatangelo et al., 2017), and chondrosarcoma (Amary et al., 2011; Borger et al., 2012; Damato et al., 2012). Inhibition of mutant *IDH*1 (m*IDH*1) reduces 2-hydroxyglutarate (2-HG) levels, which induces apoptosis and differentiation of the proliferating cancer cells.

Previous studies have identified several m*IDH*1 inhibitors with remarkable activity in preclinical models (Popovici-Muller et al., 2018). For example, BAY-1436032, developed by Bayer, is a small molecule inhibitor of m*IDH*1-R132. During Phase one of the clinal trial on acute myeloid leukemia, BAY-1436032 lacked selectivity and had susceptibility to off-target effects, which caused significant cytotoxicity, leading to the clinical trial’s termination (Chaturvedi et al., 2017; Heuser et al., 2020). Previous studies suggest that various *IDH*1 inhibitors failed to show an effect due to high mutation frequency at site 132 in *IDH* (Badur et al., 2018; Falini et al., 2019). The m*IDH*1 acquires a new function that catalyzes the production of 2-hydroxyglutaric acid (2-HG) from α-KG. 2-HG is an oncogenic metabolite that can induce tumorigenesis through various mechanisms, like affecting histone methylation, which inhibits m*IDH*1 and avoids the production of oncogenic metabolites, thus preventing tumor progression (Dang and Su, 2017).

Currently, two FDA-approved *IDH* inhibitors, enasidenib (AG-221) and ivosidenib (AG-121), are used to treat acute myeloid leukemia harboring *IDH*1 and *IDH*2 mutations. In 2019, the FDA even granted ivosidenib breakthrough therapy designation for the treatment of relapsed/refractory myelodysplasia harboring *IDH*1 mutations (Martelli et al., 2020). However, Bristol-Myers Squibb recently announced that the Phase III trial evaluating anticancer agent enasidenib (AG-221) for the treatment of *IDH*2 mutation-positive, relapsed, or refractory acute myeloid leukemia failed to meet the primary endpoint of overall survival. It is feasible and clinically beneficial to target molecularly defined solid tumors; hence, targeting *IDH*1 mutations in solid tumors is expected to become the new standard of care. Unfortunately, a vast majority of *IDH* inhibitors developed by various companies for the treatment of solid tumors have been stalled in Phase I/II of clinical trials (Supplementary Figure S1) due to poor cellular activity and selectivity or unstable pharmacokinetic properties. Hence, there is an urgent need to develop *IDH* inhibitors with novel structures and unique mechanisms of action for clinical drug use. A vast majority of available *IDH* mutation inhibitors target the catalytic site of *IDH* mutations, which are either not very active or selectively inhibit R132C mutation (Hussain et al., 2019). Therefore, developing inhibitors that target regions beyond the catalytic site is a promising approach to overcome this limitation. Recent studies have reported inhibitors that bind to various allosteric sites (Pusch et al., 2017; Machida et al., 2020) can address concerns of enzyme inhibition and effectively improve the inhibitor activity.

Structure-based virtual screening using molecular docking has become a powerful tool for hit compound discovery. This computational approach is faster and more cost-effective than experimental screening using large databases of physical compounds. (Ferreira et al., 2015; Bajad et al., 2021). Structure-based virtual screening uses the target structure as a template to screen a small-molecule database for molecules that can bind to the target. This approach can be divided into two main categories: docking-based and receptor-based pharmacophore virtual screening. By taking into consideration the effect of the receptor and ligand as a whole, molecular docking can avoid the situation of better local action and poor overall binding (Śledź and caflisch, 2018; Choudhury et al., 2022). In the current study, docking-based virtual screening and cell activity assays were used to identify compounds that target *IDH1*-R132C. ChemDiv (2019 version) database with more than 1.5 million molecules was used for the virtual screening. The compound T001-0657 was identified which showed the lowest IC_50_ (1.311 μM), and this was followed by a cell inhibition assay. The potential molecular mechanism of the newly identified compound and structural domain of *IDH1*-R132C were explored using molecular dynamics (MD) simulations and combined with free energy calculations. The identified compound T001-0657 could be a potential candidate for the treatment of cancers harboring *IDH1* mutation.

## Materials and methods

### Software

QuickVina2 is a faster and more efficient implementation of AutoDock-Vina and has a similar performance (Alhossary et al., 2015; Khalid et al., 2022); hence, QuickVina2 was used for molecular docking. A python script was used for QuickVina2, which subsequently performed a batch docking job on the small molecules in the database. The entire virtual screening process was done on a Linux operating system.

### Target preparation

The mIDH1 protein structure with co-crystallized molecular compound A (PDB: 6IO0) taht was an allosteric inhibitor was obtained from the RCSB Protein Data Bank (http://www.rcsb.org). To prepare the protein input files, protein pre-processing was carried out using SYBYL-X-2.0 software (Tripos Inc., St. Louis, MO, United States), meanwhile, all crystal water, ligands and ions were deleted. The conformation of the side-chain was optimized by searching for rotatable bonds *via* their torsion angles to release bad contacts within and between the residues and binding ligands by the use of SYBYL-X-2.0 Fix Side-chain program. Then, the polar hydrogens were added to the protein in AutoDock Tools (v1.5.6; Morris et al., 2009). The force field parameter of the protein was generated by using AMBER ff99 force field. The Gasteiger-Huckel charge was used to calculate the partial atomic charge. The number of structure optimization steps was set to 10,000, and the energy iteration was 0.005 kcal/(mol*A). The protein file was prepared and saved in pdbqt format for further analysis.

### Ligand preparation

ChemDiv, a sub-database of the ZINC15 database, was used as the virtual screening library. For QuickVina2, a total of 1,600,000 ligands were prepared as input files for docking analysis using AutoDockTools (v1.5.6; Morris et al., 2009). The OpenBabel (v2.3.1; O’Boyle et al., 2011) was then used to separate the files, and the ligands pre-processing. Finally, all the ligand files were saved in pdbqt format for the rest of the process.

### Molecular docking and virtual screening

The co-crystallized ligand structure was used to determine the binding site of the receptor protein, and AutoDock Tools (v1.5.6; Morris et al., 2009) was used to generate grid files at the centroid for ligand structure. Studies have confirmed that compound A binds to the allosteric pocket located at the dimer surface, at which is an active pocket, and the grid box and the grid box was centered on 13.052, −39.044, and 0.096 with sizes of 20 Å, 20 Å, and 20 Å. We set the molecular docking grid parameters by referring to the analysis flow of previous reports (Kumari et al., 2021), and the grid details are shown in Supplementary Table S1. Next, semi-flexible docking was then performed. The num_modes was nine, the exhaustiveness was eight, and the remaining parameters were set to the default values. In-house scripts were used for docking, and PyMOL was used for product visualizations (https://pymol.org/). 41 compounds were selected and purchased for test activity from Topscience Co.,Ltd. (TOPSCIENCE, Shanghai, China; https://www.tsbiochem.com).

### Cell culture

HT1080, U87, HT-22, 3T3, and RCTEC cells were obtained from the American Type Culture Collection Cell (ATCC, Virginia, United States). The cells were cultured as per ATCC’s instructions. The cell culture medium was supplemented with 10% (v/v) fetal bovine serum, 100 U/mL penicillin, and 100 mg/ml streptomycin (Boster, Wuhan, China). All the cells were maintained at 5%CO_2_ at 37°C.

### Cell activity test

Cells were seeded in 96-well plates and treated with the compounds (at the specific concentrations) for 48 h. DMSO was used as a control. Then, cell viability assays were performed using Cell Counting Kit-8 (Promega, Wisconsin, United States), per the manufacturer’s instructions. Each well was reconstituted with 100 μl of Cell Counting Kit-8 reagent, and the plates were shaken for 2 min for cell lysis and incubated at room temperature for 10 min 100 μl of the mixture was placed in a white 96-well luminometer plate (PerkinElmer) to measure luminescence using Envision luminometer (PerkinElmer, MA, United States). For the long-term proliferation assay, the seeding densities of the cells were determined based on the linear log-phase growth after each treatment, which was conducted in triplicates. The cells were counted, seeded at their initial seeding density, and cultured for 48 h. The IC50 values were calculated as described above.

### Molecular dynamics simulations

The Molecular Dynamics (MD) simulations for the top candidate compounds were performed using AMBER16 (Case et al., 2005) on a 100 ns timescale to investigate the stability of the docked ligand-protein complexes. The complexes were placed in a cubic box with a boundary 12 Å away from the complex, and filled with a TIP3P water model (Nayar et al., 2011). These simulated systems were prepared using the Amber ff14SB force field (Maier et al., 2015) for the protein and the gaff2 force field was used for the ligand. The Na^+^ ions were added to neutralize the system. In the first step, the geometry of all systems was minimized by the steepest descent algorithm of 2000 steps. Next, a 100 ps MD simulation was performed with force constants of 200 kcal/mol/Å^2^ under positional constraints on the C-alpha atoms and peptide ligand. The simulation time step was set to 2 fs, and the trajectory frames were recorded every 2 ps. After the initial minimization, the entire system was heated from 10 to 300 K and equilibrated for 1 ns at 300 K and 1 bar in classical (NVT) and isothermal-isobaric (NPT) combinations. The periodic boundary conditions were used in this study. Further, the particle mesh Ewald (PME) method was used to compute long-range electrostatic interactions with a cutoff of 1.2 nm, and the SHAKE algorithm was used to constrain the motion of the hydrogen bonds. Finally, a 100 ns timescale MD simulation was performed and repeated three times. The trajectory exploration, including root-mean-square deviation (RMSD) and root-mean-square fluctuation (RMSF) was performed using the PTRAJ (process trajectory) and CPPTRAJ modules in AMBER16.

### Binding free energy and per-residue decomposition studies

The MMGBSA. py program, implemented in AMBER16, was used to calculate the binding free energies. The molecular mechanics Poisson−Boltzmann surface area (MM-GBSA) method was used to calculate the binding free energy (ΔG_bind_) and per-residue decomposition analysis based on 1,000 snapshots extracted from the last 10,000 frames after equilibration at a time interval of 10 ps. The ΔG_bind_ calculated using the MMGBSA method is summarized in Eqs 1–4. (Gohlke and Case., 2004). The energy contribution of each residue in each system was also examined using residue-specific energy decomposition. All the other parameters were kept as the default value. Additionally, -TΔS represented the contribution of the entropy of solute molecules. Because the calculation of entropy was computationally expensive for large systems and had the tendency to introduce low-accuracy approximations, -TΔS was not considered in the present work (Miller et al., 2012).

$$\Delta {\text{G}}_{\text{bind}}=\Delta {\text{G}}_{\text{complex}}-\Delta {\text{G}}_{\text{receptor}}-\Delta {\text{G}}_{\text{ligand}}$$ (1)

$$\Delta {\text{G}}_{\text{bind}}=\Delta {\text{E}}_{\text{MM}}+\Delta {\text{G}}_{\text{solv}}-T\Delta S$$ (2)

$$\Delta {\text{E}}_{\text{MM}}=\Delta E_{\text{vdw}}+\Delta E_{\text{ele}}+\Delta E_{\text{intra}}$$ (3)

$$\Delta G_{\text{solv}}=\Delta G_{\text{GB}}+\Delta G_{\text{SA}}$$ (4)

## Results and discussion

### Structure-based virtual screening and preliminary biological screening

**FIGURE 1:**
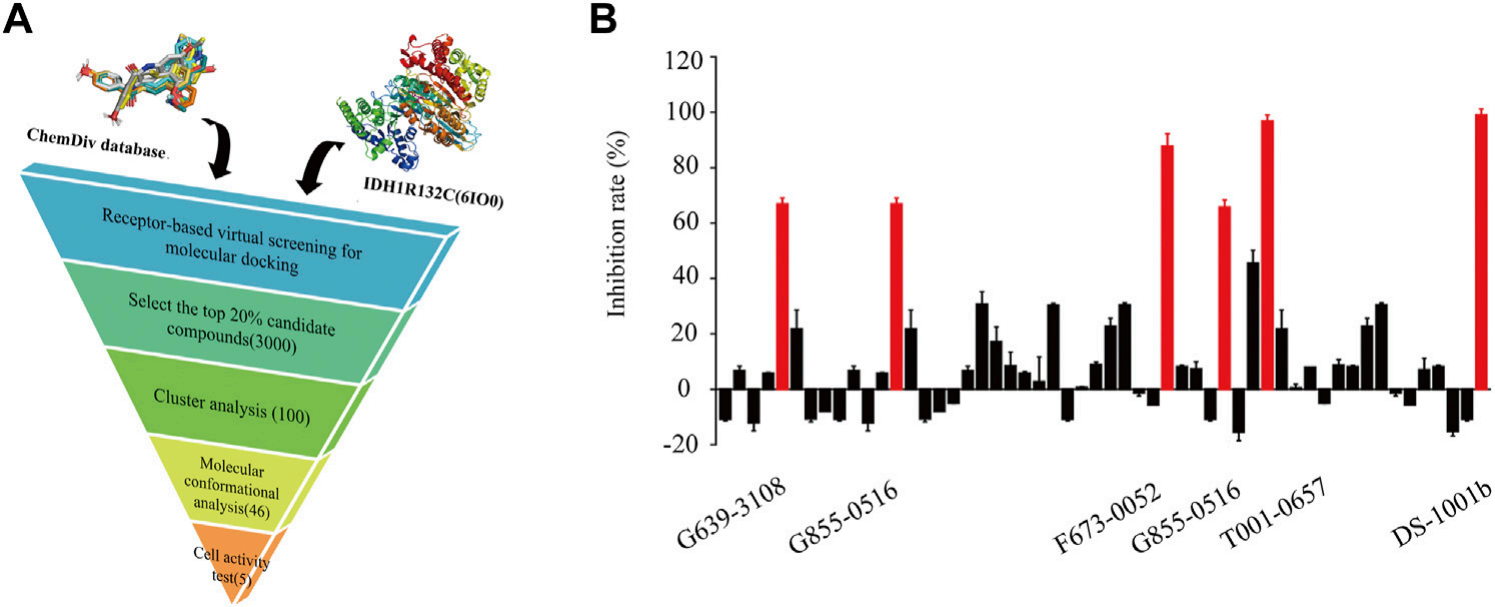
Identification of potential IDH1-R132C inhibitors. **(A)** Flowchart of the hit discovery using docking-based virtual screening to discover of *IDH1*-R132C inhibitors. **(B)** The corresponding inhibition rate of 46 molecules at 10 μM in the HT1080 cells.

**FIGURE 2:**
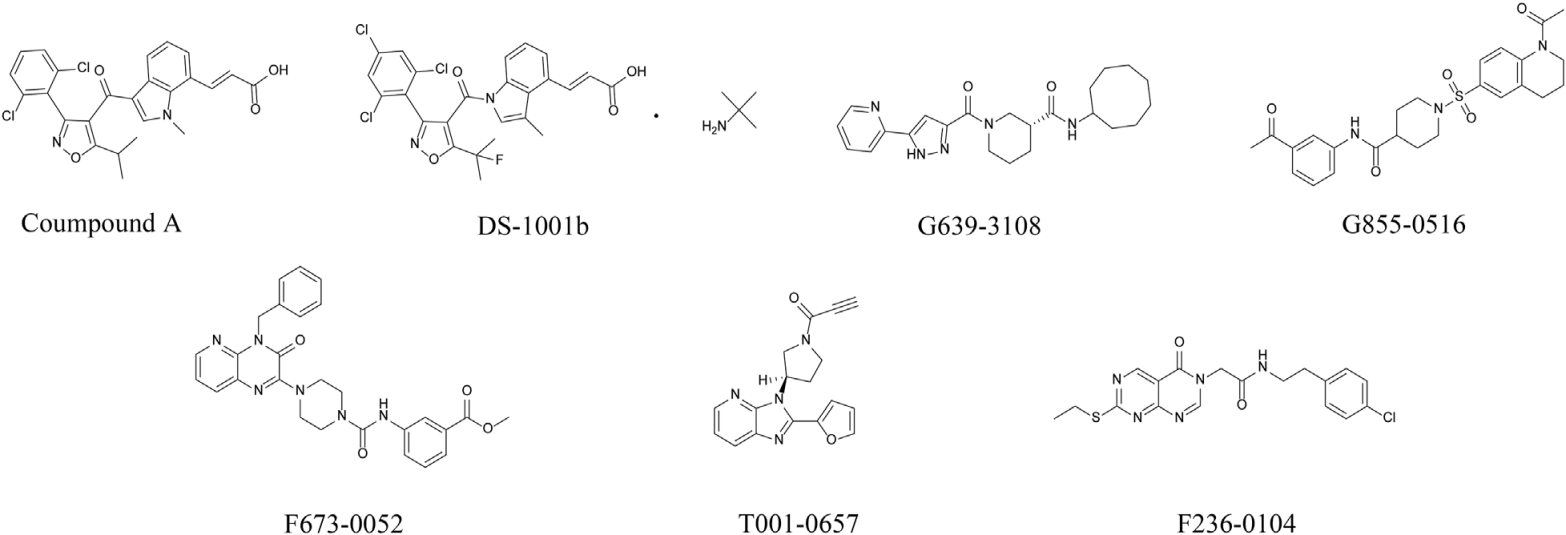
Chemical structures of the positive compound and five candidate compounds after preliminary cellular evaluation. (Error bars are mean ± S.D. for three replicates).

Figure 1A shows the workflow of the structure-based virtual screening. Before performing virtual screening, the eutectic molecule compound A of 6IO0 was docked into its active pocket and a binding energy of −8.9 kcal/mol was obtained. The scoring value of compound A was used as a control for the virtual screening. After the docking was completed, the screened compounds were filtered through the pan-assay interference compounds filter. The docking scoring data corresponding to the filtered small molecules were subsequently extracted for sorting, and 3,000 small molecules with scoring values below −8.9 kcal/mol were selected. Based on the binary fingerprints, the k-means clustering method in Canvas 2.3 was used to cluster the 3,000 compounds into 100 classes. These 100 compounds were then analyzed for conformation and ease of later modification, and finally, 46 compounds were identified and purchased to preliminarily evaluate the inhibitory effects on the HT1080 cells harboring *IDH1*-R132C mutation. Five compounds, including G639-618, G855-0516, F673-0052, T001-0657, and F236-0104, showed positive results in the CCK-8 assay and showed >50% inhibition on the HT1080 cells at a concentration of 10 μM (Figure 1B). Figure 2 shows the molecular architectures of the five compounds, the physicochemical attributes and docking scores of the five compounds are shown in Table 1. Furthermore, Table 2 lists the ADMET values for the five compounds. Supplementary Table S2 summarizes the docking scores and physical characteristics of the rest of the 41 selected compounds.

**TABLE 1:** Docking scores, physicochemical properties, and IDH1-R132C inhibition activities of the screened compounds.

Compound	Docking Score(kcal/mol)	MW	HBD	HBA	ROB	HA	LogPo/w	TPSA	Inhibition (%; 10 μM)	LogD	Water solubility	Formal charges	rings	The maximum size of rings	Rotatable bonds	SAscore	stereocenters
DS-1001b	−8.9	485.36	1	5	6	33	3.3	83.64	100.41	3.382	2.34e-05 mg/ml	0	4	9	6	3.233	0
compound A	−8.9	483.34	1	5	6	33	3.33	85.33	—	3.856	1.34e-04 mg/ml	0	4	9	6	2.915	0
G639-3,108	−9.3	409.52	2	4	6	30	2.86	90.98	64.87	3.464	1.90e-02 mg/ml	0	4	8	6	2.879	1
G855-0516	−9.6	483.58	1	6	7	34	2.98	112.24	66.13	2.321	7.84e-02 mg/ml	0	4	10	7	2.309	0
F673-0052	−9.1	498.53	1	6	8	37	3.86	109.66	88.75	3.233	3.16e-02 mg/ml	0	5	10	8	2.345	0
T001-0657	−9	306.33	0	4	3	23	2.86	64.16	60.23	1.076	3.34e-01 mg/ml	1	4	9	3	4.005	1
F236-0104	−8.9	403.89	1	5	8	27	2.43	115.07	99.35	2.761	4.88e-02 mg/ml	0	3	10	8	2.425	0

**TABLE 2:** The ADMET values of the screened compounds.

Compound	HIA	BBB penetration	CYP2D6 inhibitor	T_1/2_	hERG blockers
DS-1001b	−−−	−−	−	0.034	−−−
compound A	−−−	−−	−	0.015	−−−
G639-3,108	−−−	−−	−−−	0.137	−−
G855-0516	−−−	++	+	0.203	−−
F673-0052	−−−	−−	−	0.399	+++
T001-0657	−−	+++	−−−	0.753	−−
F236-0104	−−−	+++	−	0.541	−−

HIA, Human Intestinal Absorption; BBB, Penetration: Blood-Brain Barrier Penetration; CYP2D6, CYP450 inhibitor; T_1/2_ hERG Blockers, Drug Cardiotoxicity Prediction. 0–0.1(−−−), 0.1–0.3(−−), 0.3–0.5(−), 0.5–0.7(+), 0.7–0.9(++), 0.9–1.0(+++). 0–0.3:good; 0.3–0.7:medium; 0.7–1.0:bad.

### Cell proliferation assays

**FIGURE 3:**
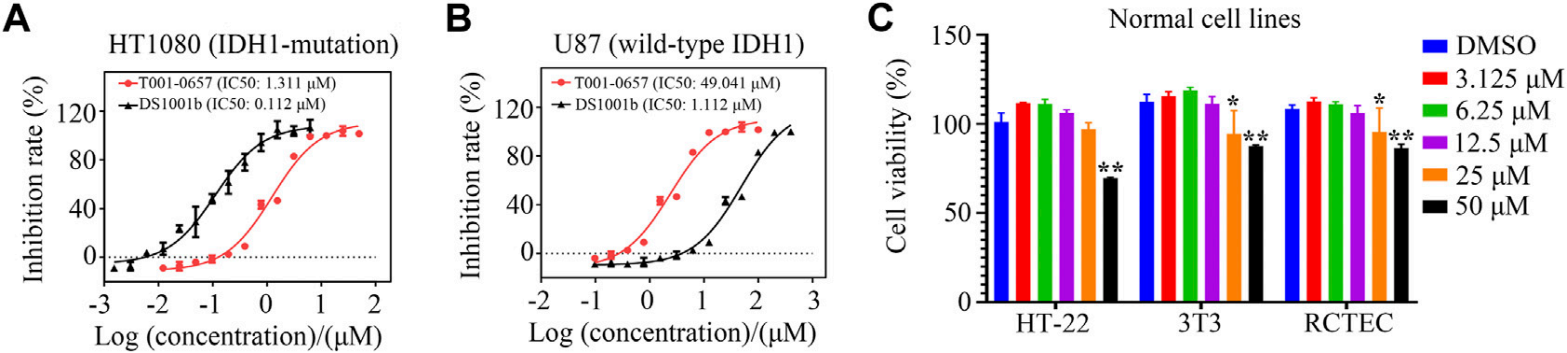
T001-0657 suppresses IDH1 activity in vitro. **(A)** T001-0657 inhibits the HT1080 cell proliferation, with an IC_50_ value of 1.311 μM. **(B)** T001-0657 inhibits the proliferation of U87 cells with an IC_50_ value of 49.041 μM. **(C)** Normal cells were exposed to various concentrations of T001-0657 for 48 h to determine the cytotoxic activity. (Each point represents the mean ± standard deviation of the three replicates). Significance: *, *p* < 0.05; **, *p* < 0.01.

To test whether T001-0657 specifically inhibited cancer cell proliferation, we used the HT1080 cell’s harboring *IDH1*-R132C mutation and the U87 MG cell line, which has wild-type *IDH1*. A proliferation assay was performed to determine the cellular activity of T001-0657. The IC_50_ value for the positive compound DS1001b was 0.112 μM, which is consistent with previous reports (Machida et al., 2020), thus verifying the reliability of this cellular assay. The results reveal that T001-0657 was potent and inhibited cell proliferation in a dose-dependent manner. Further, T001-0657 had high inhibitory effect on HT-1080 cells, with an IC_50_ value of 1.311 μM (Figure 3A). Interestingly, the IC_50_ value of T001-0657 was 49.041 μM for the U87 cells (Figure 3B). As shown in Figure 3C, T001-0657 had minimal inhibitory effect on the proliferation (IC_50_ > 50 μM) on the proliferation of normal human cells (HT-22, 3T3, and RCTEC). These results suggest that T001-0657 may have a highly selective inhibitory effect on cancer cells harboring *IDH1*-R132C mutation but not on normal cells and cancer cells with the wild-type *IDH*1. Our results show that T001-0657 had satisfactory potency and low toxicity. It is a promising lead compound for the prevention and treatment of cancers associated with *IDH1* mutations.

### Docking studies and interaction analysis

The *IDH*1 gene has three domains: a large domain (residues 1–103 and 286–414), small domain (residues 104–136 and 186–285), and a clasp domain (residues 137–185). Tyr139 may play a critical role in the reduction of α-KG to 2-HG by compensating for the increased negative charge on the C2 atom of α-KG in the intermediate state (Yang et al., 2010). Owing to the strong polarity of amino acids around the pocket, Tyr139 is crucial for catalysis. The compound A does not interact with Tyr139 but binds to the allosteric active pocket of *IDH1*-R132C.

**FIGURE 4:**
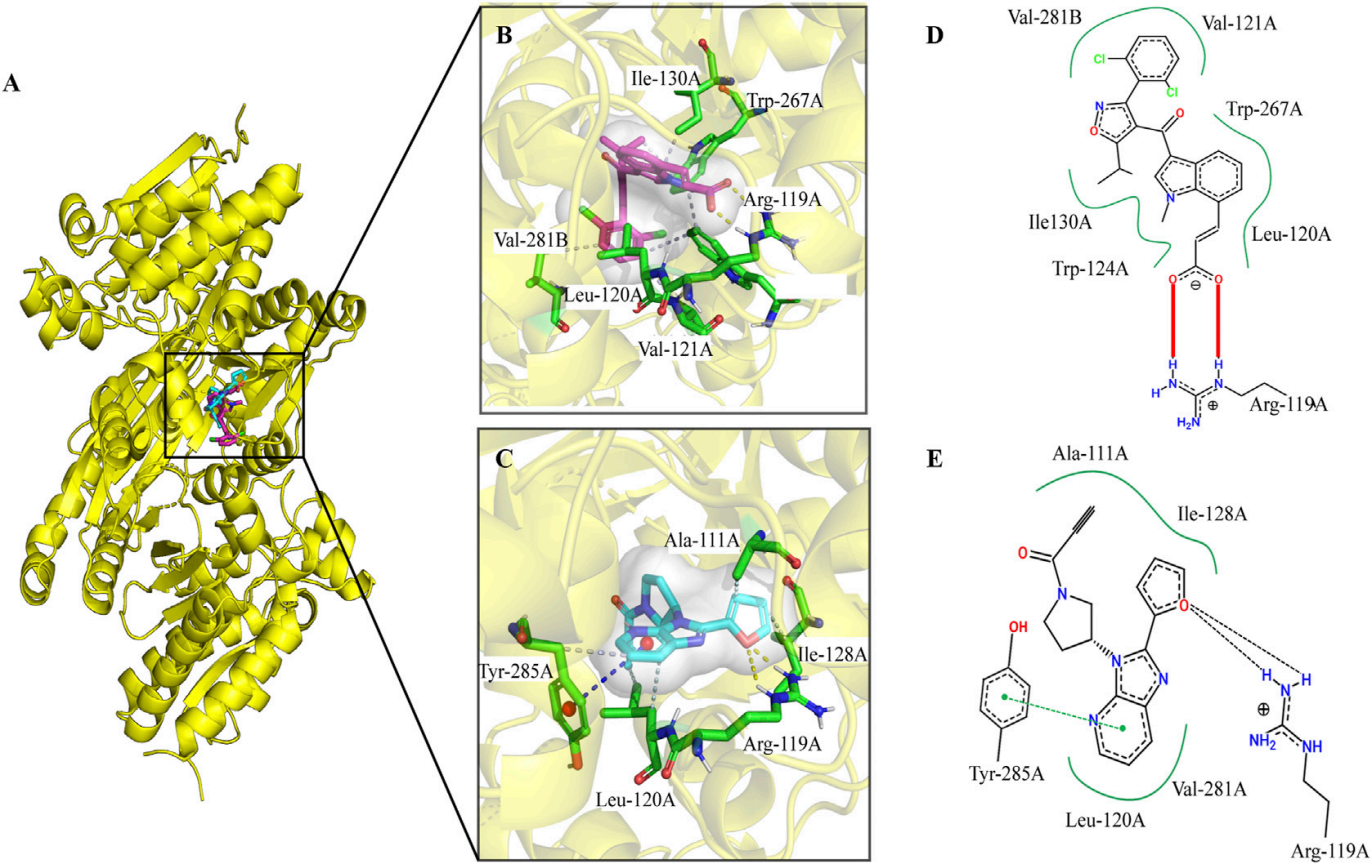
The binding pocket of compound A and T001-0657 for IDH1-R132C. **(A)** Overall figures of the binding of compound A and T001-0657 to *IDH1*-R132C. Compound A is depicted as red sticks and T001-0657 is depicted as blue sticks. The *IDH1-*R132C is displayed in cartoon mode (PDB ID 6IO0). **(B,C)** A close-up view of the key interactions stabilizing compound A/T001-0657 in the *IDH1*-R132C binding pocket. Compound A/T001-0657 is depicted as red/blue sticks, the surrounding key residues are shown as green sticks and labeled. The hydrogen bonds are shown as a yellow dashed line. **(D,E)** Two-dimensional binding mode diagram of compound A and T001-0657 with *IDH1*-R132C; the red solid line indicates the salt-bridge interaction, the green dashed line indicates the π-π stacking interaction, and the black dashed line indicates the hydrogen bonding interaction.

The docking poses generated by QuickVina2 were analyzed to determine the binding mode of the *IDH1* inhibitors. To reveal the interaction model and to inhibit the mechanism between lead compound A and m*IDH1*, compound A was redocked to the allosteric binding site of 6IO0, and the interactions were then analyzed. According to a previous report, compound A binds to the allosteric pocket located at the dimer surface, and the residues in the allosteric pocket form an α/β sandwich structure (Xu et al., 2004). This conformational change disrupts the spatial arrangement of the Asp residues (Asp275, Asp279, and Asp252 in another protomer), which form the binding site for a catalytically important divalent cation. This conformational change reduces the affinity for the substrate α-KG because the coordinate bond formation with the divalent cation was necessary for α-KG binding. As a result, compound A alters the overall catalytic activity of the *IDH1* mutants by lowering the binding affinity of the divalent cations and substrate. Based on the analysis of the binding modes of the two inhibitors, the binding sites of compound T001-0657 and compound A largely overlapped, (Figure 4A), and both the compounds interacted with Arg119. Figure 4B shows that compound A stably bound to the hydrophobic cavity formed by Val281, Val121, Trp267, Leu120, Ile130, and Trp124. Further, a salt bridge was formed between the carboxyl group and Arg119, corroborated with the two-dimensional diagram in Figure 4D, where the entire molecular structure is wrapped in a binding pocket. A previous study showed that compound A showed inhibitory activity against *IDH1*-R132C (IC_50_ = 130 nmol/L) (Machida et al., 2020). Regarding the docked binding mode of T001-0657 with *IDH1*-R132C, the docking structure of *IDH1*-R132C with T001-0657 showed that compound T001-0657 formed a critical hydrophobic interaction with Ala111, Ile128, and Leu120, and with two hydrogen bonds between the furan ring and Arg119 (Figure 4C). In addition, stable π-π stacking (T-stacking) was identified between the pyridine of compound T001-0657 and the benzene ring of Tyr285, which is a weak aromatic interaction and identifies the importance of the conformational stability of the ligand, as illustrated by the two-dimensional interaction pattern diagram in Figure 4E. The T001-0657 structure was biased toward the carboxyl group of compound A, and thus lacked hydrophobic interactions with amino acids ILE130, TRP267, VAL255, and VAL281B. Secondly, both compound A and T001-0657 formed two hydrogen bonds with ARG119, but the hydrogen bond with ARG119 was formed by the carboxyl part of compound A, which was stronger than that of T001-0657. Finally, the pyridine ring of T001-0657 had a stable t-π stacking interaction with TYR285. These results were the main cause of the difference in activity between compoundA and T001-0657.

### Stability of the *IDH1* inhibitor system

**FIGURE 5:**
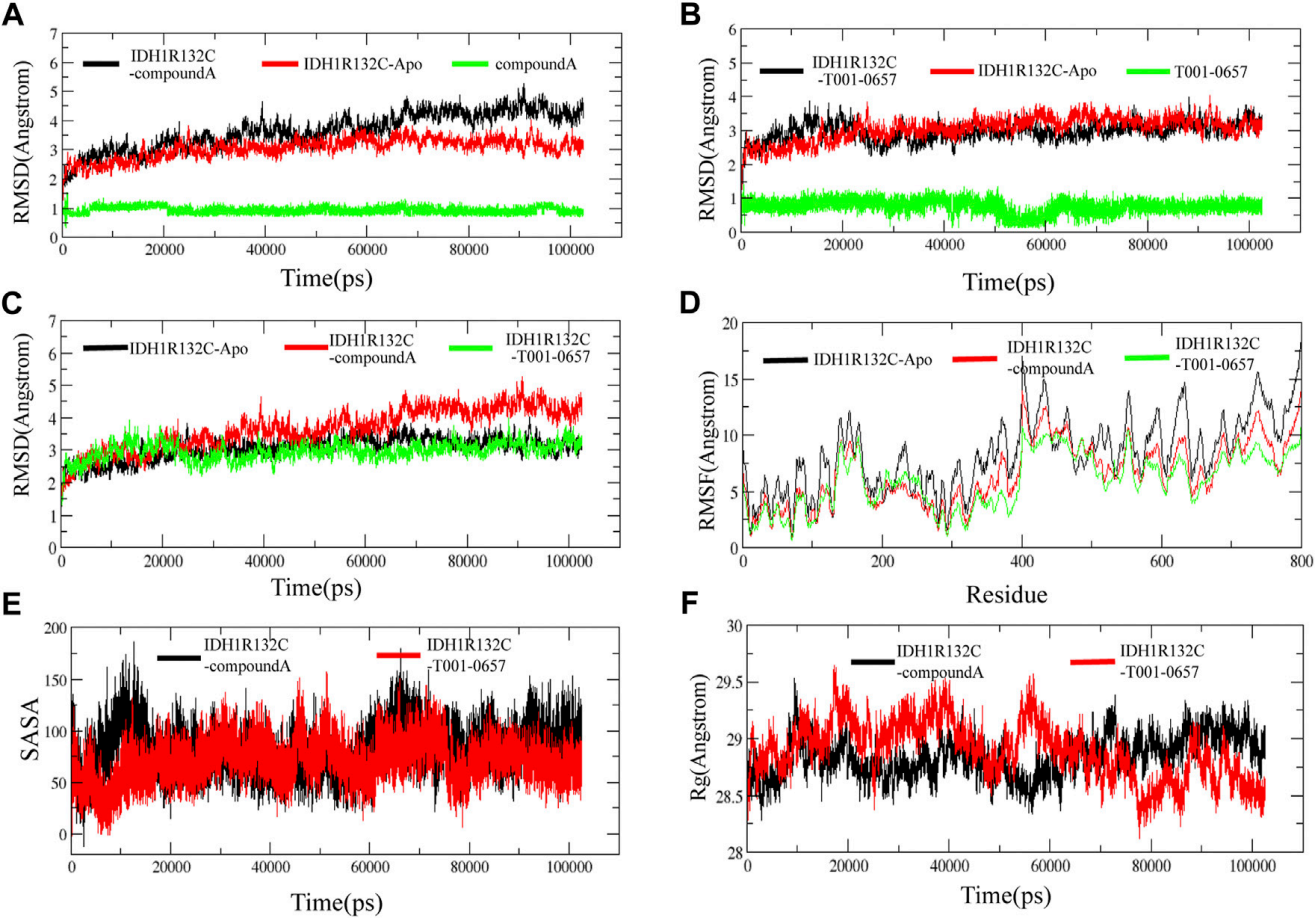
Stability of the IDH1 inhibitor system. **(A)** Root-mean-square deviation (RMSD) of the backbone atom of *IDH*1-R132C, the complex formed by *IDH*1-R132C with compound A and the heavy atom of compound A. **(B)**The RMSD of the backbone atom of *IDH*1-R132C, the complex formed by *IDH*1-R132C with T001-0657 and the heavy atom of T001-0657. **(C)** The RMSD of the backbone atom of *IDH1*-R132C, the complex formed by *IDH1*-R132C with compound A, and the complex formed by *IDH1*-R132C with T001-0657. **(D)** The root-mean-square fluctuation of the backbone atom of *IDH1*-R132C and *IDH1*-R132C in the complex with compound A and T001-0657. **(E)** The solvent-accessible surface area analysis of compound A and T001-0657 bound with *IDH1*-R132C. **(F)** The mass-weighted radius of gyration.

To identify the stability of these complexes, 100 ns MD simulations were performed for compound A and the T001-0657-*IDH1*-R132C complexes identified from our virtual screening studies. The RMSD values of the *IDH1*-R132C complex, the backbone atoms of *IDH1*-R132C, and the ligand were calculated using the MD simulation. RMSD values can measure if the simulated system reached equilibrium, and lower RMSD values indicate a more stable protein complex. The RMSD plot (Figure 5A) shows that the simulation of the *IDH1*-R132C-compound A complex system proceeded to 70 ns before reaching convergence, and Figure 5B shows that the simulation of the *IDH1*-R132C-T001-0657 complex system proceeded to 60 ns before reaching convergence. The average RMSD value of *IDH1*-R132C-compound A was 3.71 Å and for *IDH1*-R132C-T001-0657 it was 3.10 Å (Table 3). All the systems reached equilibrium after 100 ns of MD simulation (Figure 5C), indicating that compound A and T001-0657 can bind stably to *IDH1*-R132C. These results confirm that all the simulated systems reached a stable state during MD simulations. The RMSDs of compound A and T001-0657 to *IDH1*-R132C in the three MD simulations are shown in Supplementary Figure S2. The RMSFs of the Cα-atoms in both complexes were calculated to check the residue flexibility during the MD simulations. As shown in Table 3, the RMSFs of the *IDH1*-R132C-drugs complexes and *IDH1*-R132C-apo were in the range of 5.96 and 8.34 Å, respectively. Further, all the RMSFs of the *IDH1*-R132C-drugs complexes were lower than that of *IDH1*-R132C-apo (Figure 5D).

**TABLE 3:** The average root-mean-square deviation, root-mean-square fluctuation, radius of gyration, and solvent-accessible surface area of the IDH1-R132C-apo, IDH1-R132C-compound A and T001-0657 complexes.

Complexes	Average RMSD (Å)	Average RMSF (Å)	Average RoG (Å)	Average SASA (Å^2^)
IDH1R132C-Apo	2.84	8.34		
IDH1R132C-compoundA	3.71	6.63	28.97	79.16
IDH1R132C-T001-0657	3.10	5.96	28.92	70.74

In solution, hydrophobic interactions between the nonpolar amino acids are essential for the formation of stable hydrophobic binding sites in proteins. In this study, the stability of the two proteins in solution was analyzed by calculating the solvent-accessible surface area (SASA) of the two ligands, compound A and T001-0657, bound to *IDH1*-R132C. As shown in Figure 5E, the SASA of compound A bound to *IDH1*-R132C fluctuated dynamically during the simulation for 20 ns. In the SASA of T001-0657 combined with *IDH1*-R132C, T001-0657 also fluctuated for 20 ns, however the SASA decreased significantly compared to that of compound A, and the two ligands maintained the same trend during the next simulation. These results indicated that compound A and T001-0657 combined with *IDH1*-R132C could maintain the stability of *IDH1*-R132C in solution, and these results are consistent with these results of the RMSD.

The radii of gyration describe the distribution of the system atoms along a specific axial direction and can be used to characterize the closeness of the molecules. As shown in Figure 5F, after 100 ns of simulation, the radii of gyration of both compound A and T001-0657 ligands were stable, and there was no significant difference between their height and amplitude. The average Rg values of compound A was 28.97, and T001-0657 was 28.92 Å (Table 3). These results indicate that the *IDH1*-R132C-compound A and the *IDH1*-R132C-T001-0657 complexes, became more compact due to the binding of the ligands, thereby reaching a stable state. The average RMSD, RMSF, Rg, and SASA of the three MD simulations of compound A and T001-0657 are shown in Supplementary Table S3.

Further, the distribution and number of hydrogen bonds in the complexes were investigated to determine the stability of the system during the simulation. The intramolecular hydrogen bond plot and distribution of the hydrogen bond lengths show that compound T001-0657 had hydrogen bonds comparable to compound A (Supplementary Figure S3). Overall, the hydrogen bonding results indicate that the binding of the ligands to *IDH1*-R132C formed a stable complexes.

Finally, we compared the snapshots of the protein-ligand complexes at different time intervals of the MD simulation to evaluate the stability of the ligands with proteins during the MD simulations. The results showed that the selected conformations remained stable throughout the MD simulations (Supplementary Figures S4, S5). The residue contact_maps in different simulated systems were shown in Supplementary Figures S6A–C, and it could be seen that IDH1R132C-compound A and IDH1R132C-T001-0657 complexes had similar contact_map during the MD simulation. Supplementary Figures S6D,E showed the native contact between the ligand and the residues, which indicated that the residue contact regions was generally consistent with those in the snapshots of protein-ligand complexes at different time intervals of molecular dynamics (Supplementary Figures S5, S6). The darker color in the thermogram indicates a higher relative contact strength during the MD simulation. For the IDH1R132C-compound A system, Leu120, Trp124, Ile128, Ile130, Trp267, Gln277, Ser278, and Gln273(B) showed greater than 50% relative contact strength during the MD simulation and were the key residues for compound A binding to IDH1R132C; while for T001-0657, the key residues were mainly Arg119, Leu120, Trp124, and Leu128, which showed greater than 50% relative contact strength, and Ser267 and Val281 also showed 45.97% and 42.75% relative contact strength, respectively.

Hence our analysis shows that, each simulation system has reached a stable state during the simulation.

### Binding free energy and per-residue decomposition studies

The ΔG_bind_ of compound A and T001-0657 that was calculated using the MM-GBSA method to evaluate the binding differences for compound A and T001-0657. As shown in Table 4, the average ΔG_bind_ for R132C-compound A and R132C-T001-0657 was −42.58 kcal/mol and −30.62 kcal/mol, respectively, which is consistent with the order of the experimental IC_50_ values (Supplementary Table S4). As mentioned above, the experimental IC_50_ value for compound A was 130 nmol/L (Machida et al., 2020), while it was 1.311 μmol/L for T001-0657. The Van der Waals interaction contribution (ΔG_vdw_) was the most important factor for the ΔG_bind_ of each complex. The benzene ring structures of 1,3-dichlorobenzene and the indole ring in compound A formed hydrophobic interactions with Leu120, Val121, Trp124, Ile130, Trp267, and Val281, which allowed compound A to occupy the highest Van der Waals interaction energy (-57.68 kcal/mol), while in the R132C-T001-0657 system, the pyridine and furan rings of T001-0657 formed hydrophobic interactions with Val281, Leu120, Ile128, and Ala111 (−43.92 kcal/mol). Additionally, the electrostatic contribution (ΔG_ele_) was also important to the ΔG_bind_ of each complex, which was in agreement with the results of the MD simulations. The unfavorable polar solvation contribution (ΔG_GB_) significantly affected the ΔG_bind_, indicating the main differences between the two complexes. Furthermore, the favorable nonpolar solvation contributions (ΔG_surf_) to the ΔG_bind_ of each complex were similar (Supplementary Figure S7).

**TABLE 4:** The binding free energy contributions of compound A and T001-0657 to IDH1-R132C calculated using the MM-GBSA method (kcal/mol).

Complexs	Contributions							ΔG_avg_
	ΔE_vdw_	ΔE_ele_	ΔE_GB_	ΔE_surf_	ΔE_MM_	ΔE_sol_	ΔG_bind_		
compoundA	−54.61 ± 0.28	−10.51 ± 0.34	29.00 ± 0.21	−4.87 ± 0.01	−65.12 ± 0.33	24.13 ± 0.21	−40.99 ± 0.25	−42.58 ± 0.26
	−57.68 ± 0.34	−15.12 ± 0.29	33.95 ± 0.24	−5.09 ± 0.03	−72.80 ± 0.51	28.86 ± 0.23	−43.94 ± 0.42	
	−55.75 ± 0.08	−12.38 ± 0.09	30.20 ± 0.07	−4.88 ± 0.01	−68.13 ± 0.12	25.32 ± 0.07	−42.81 ± 0.10	
T001-0657	−44.71 ± 0.07	−11.41 ± 0.11	28.59 ± 0.09	−3.72 ± 0.00	−56.11 ± 0.12	24.87 ± 0.09	−31.25 ± 0.08	−30.62 ± 0.22
	−44.61 ± 0.20	−12.46 ± 0.37	29.72 ± 0.29	−3.74 ± 0.01	−57.07 ± 0.38	25.99 ± 0.28	−31.08 ± 0.24	
	−42.44 ± 0.25	−8.85 ± 0.52	25.78 ± 0.33	−4.01 ± 0.01	−51.30 ± 0.52	21.77 ± 0.33	−29.53 ± 0.33	

**FIGURE 6:**
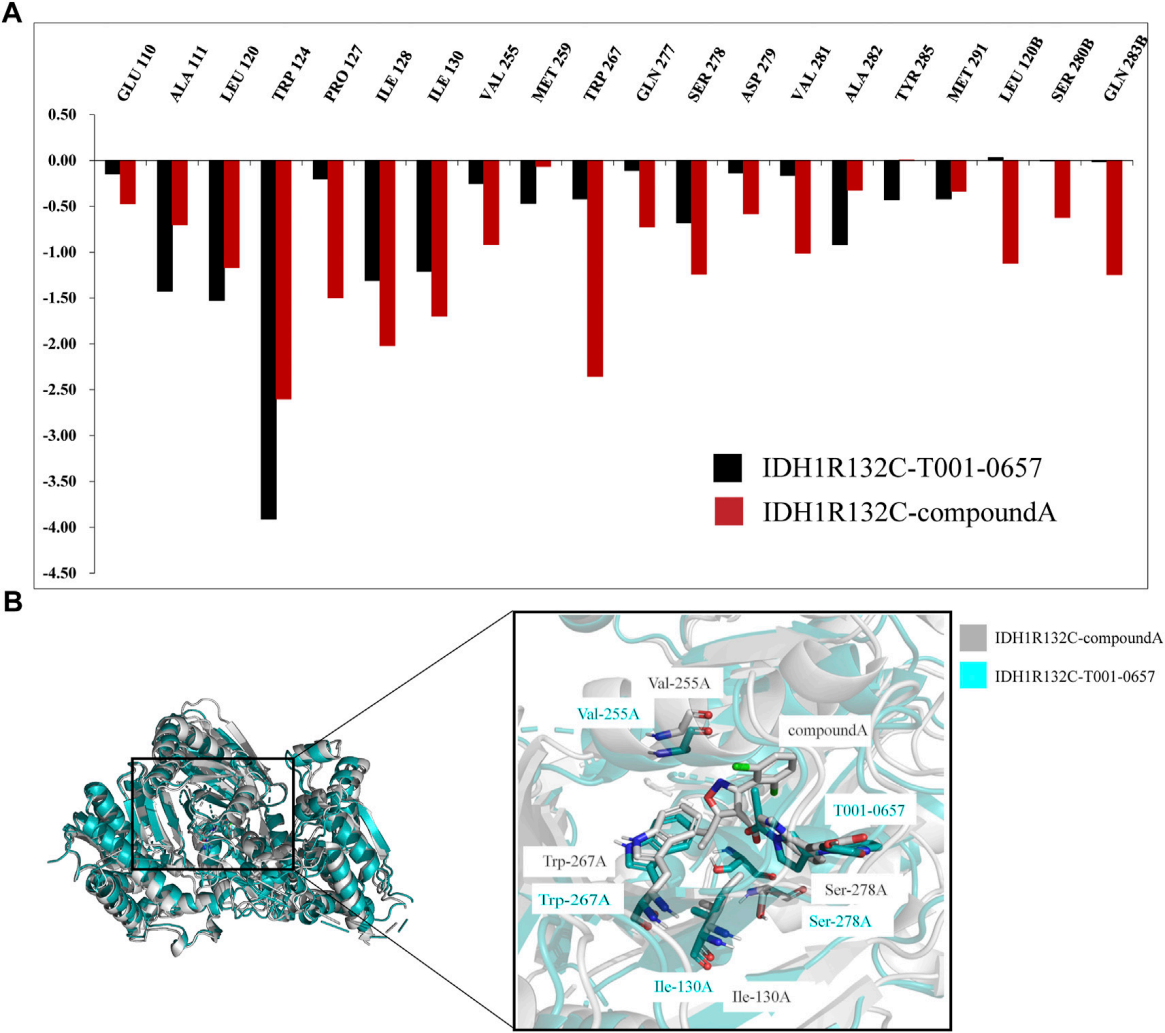
Binding free energy and per-residue decomposition studies. **(A)** A comparison of the specific energy contributions of *IDH1*-R132C inhibitors bound to key residues. **(B)** A comparison of the average conformational overlap maps generated by extracting 1,000 frames of conformation from the two complex traces.

To analyze the contribution of the interacting residues to the binding of the inhibitors to *IDH1*-R132C, the per-residue decomposition of the *IDH1*-R132C complexes was determined. Figure 6A shows residues with energy contributions greater than 0.5 kcal/mol, which is considered as key residues. The specific energy contribution values of the two compounds with *IDH1*-R132C are summarized in Supplementary Table S5.

Seven key residues, Trp124, Ile128, Ile130, Val255, Trp267, Ser278, and Val281, were identified in *IDH1*-R132C-compound A. The key residues Pro127, Ile130, Val255, Trp267, and Ser278 were identified as the main differences between compound A and T001-0657 when binding with *IDH1*-R132C. When compound A bound to *IDH1*-R132C, the energy contributions of the five key residues mentioned above were −1.50 kcal/mol, −1.69 kcal/mol, −0.91 kcal/mol, −2.35 kcal/mol, and −1.24 kcal/mol, respectively. However, with T001-0657, the favorable free energy contribution was transformed into a relatively less favorable contribution as follows: Pro127: −0.20 kcal/mol, Ile130: −1.21 kcal/mol, Val255: −0.25 kcal/mol, Trp267: −0.42 kcal/mol, and Ser278: −0.68 kcal/mol. This was the main reason for the difference in activity between the two groups. To visualize the effect of these four key amino acids on the binding pattern (Figure 6B), we extracted two composite systems, *IDH1*-R132C-T001-0657 and *IDH1*-R132C-compound A, which maintained a stable 1000-frame conformation during the simulation, generated an average conformation, and superimposed the generated average conformation to observe the conformational differences between the two active sites. The superimposed pattern of T001-0657 and compound A showed that the structure of T001-0657 was biased towards the carboxyl group of compound A. This resulted in weaker hydrophobic interactions and lower energy contribution values for Pro127, Ile130, Val255, and Trp267. The key amino acid Ser278 showed a large positional shift in the binding of the ligand to the active pocket, which also led to a decrease in the energy contribution.

## Conclusion

In summary, the study identified a potent *IDH1*-R132C inhibitor based on molecular docking-based virtual screening using the ChemDiv database and cellular assays. The MD simulations and ΔG_bind_ calculations showed that nonpolar interactions were the dominant force that led to binding the inhibitor to *IDH1*-R132C. Additionally, the pyridine ring of T001-0657 and the π-π stacking interaction formed by the benzene ring of Tyr285 contributed favorably to the binding energy. The H-bond between the inhibitor and Arg119 played a key role in the inhibitory activity of the compounds. Thus, enhanced hydrogen bonding and nonpolar interactions between *IDH1*-R132C and the inhibitor will contribute to further structural optimization. Therefore, T001-0657 is a powerful inhibitor that could be used to further investigate the biological role of *IDH1*-R132C and has the potential to provide therapeutic strategies for a wide range of cancer indications.

## Data Availability

The original contributions presented in the study are included in the article/Supplementary Materials, further inquiries can be directed to the corresponding authors.
