# Supplementary material for: Discovery of novel IDH1-R132C inhibitors through structure-based virtual screening
Source: Front Pharmacol. 2022 Sep 7;13:982375. doi: 10.3389/fphar.2022.982375 (PMC9491111; doi:10.3389/fphar.2022.982375)
Supplement: Supplementary file 1 [file DataSheet1.docx]

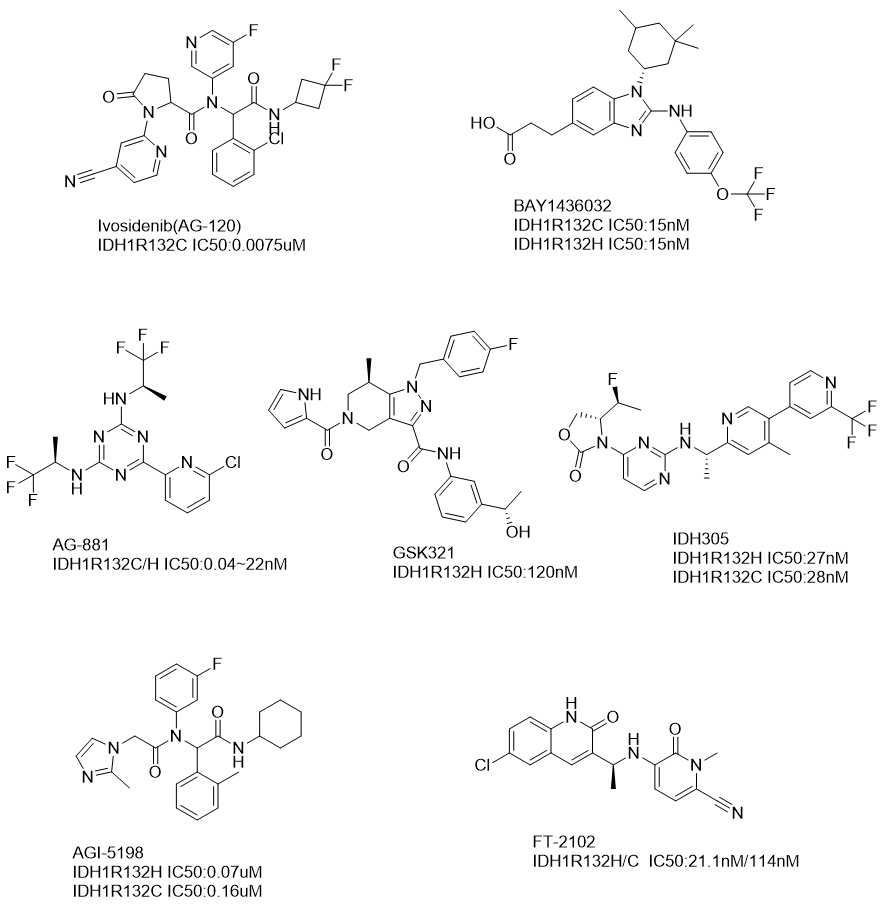


**Supplement Figure 1. IDH inhibitors that have entered clinical studies**


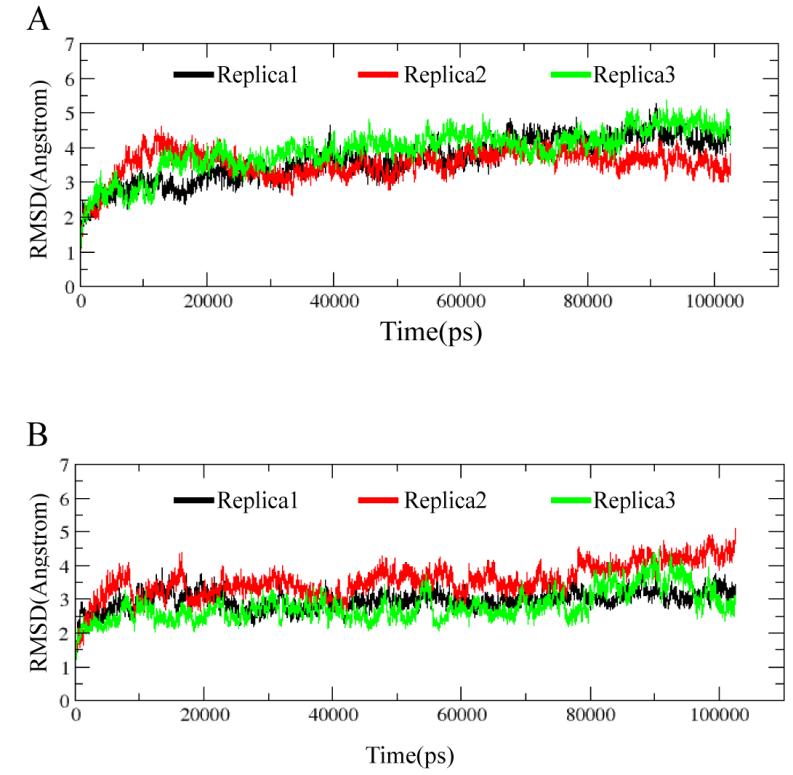


**Supplement Figure 2. Root-mean-square deviations (RMSDs) of complexs of three replicas: A:IDH1R132C-compoundA; B:IDH1R132C-T001-0657.**

**
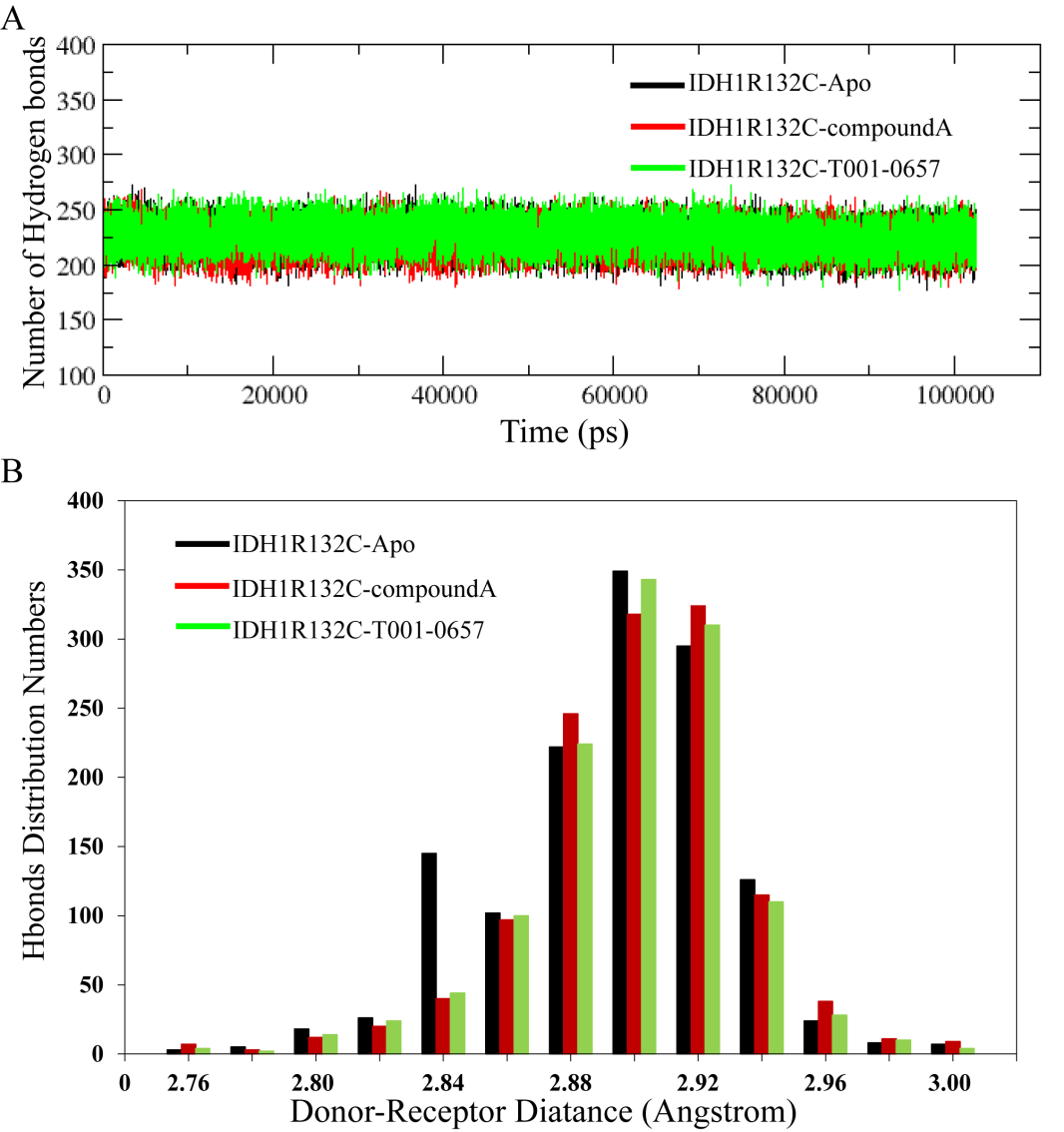
**

**Supplement Figure 3. Intra hydrogen bond numbers (A) and distribution patterns (B) for IDH1R132C-compoundA and IDH1R132C-T001-0657 complexes for the molecular dynamics of 100 ns at 300 K.**

**
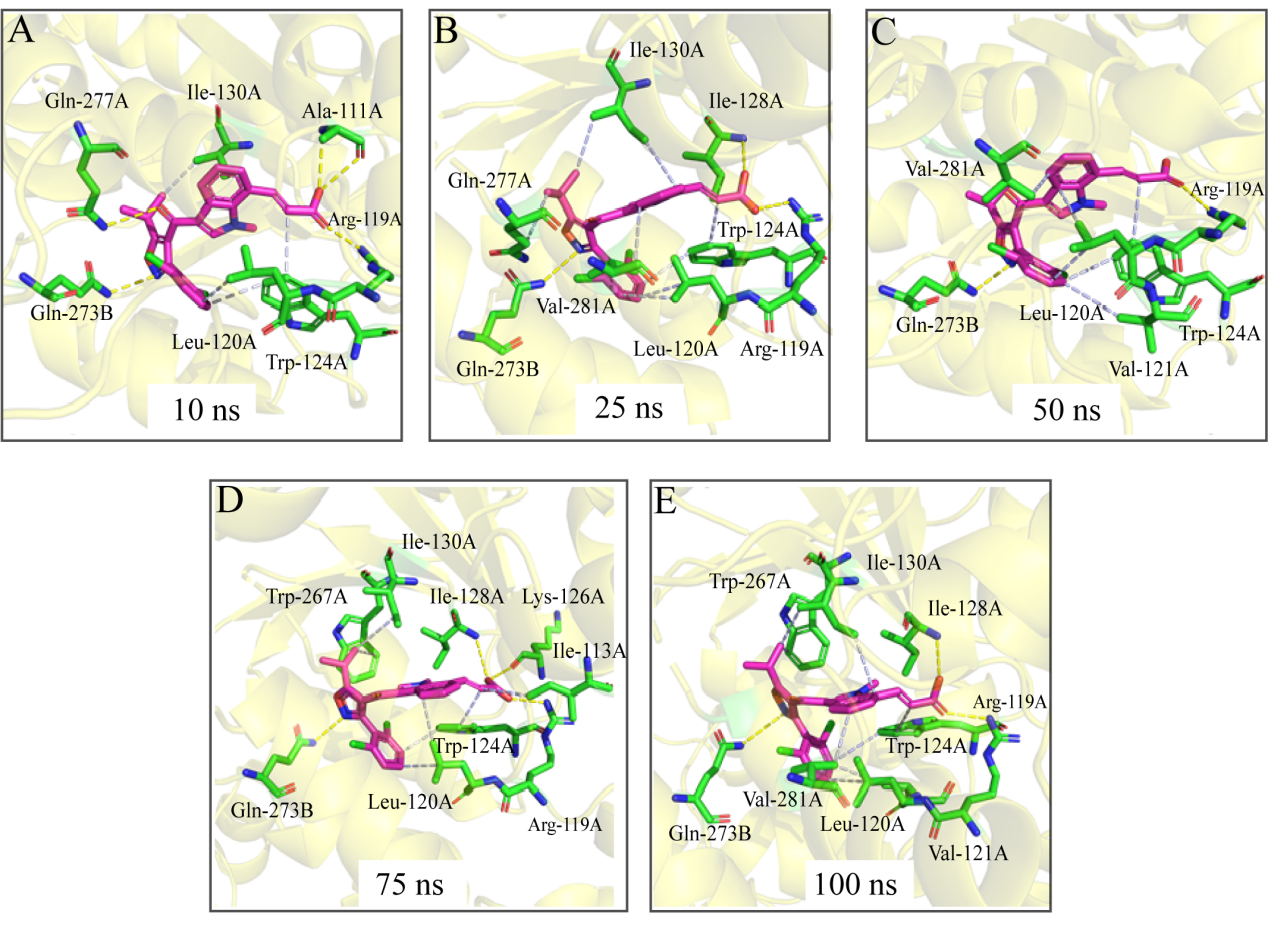
**

**Supplement Figure 4. Conformations of IDH1R132C-compoundA complex at different stages of MD simulation: (a) 10 ns, (b) 25 ns, (c) 50 ns, (d) 75 ns and (e) 100 ns.**


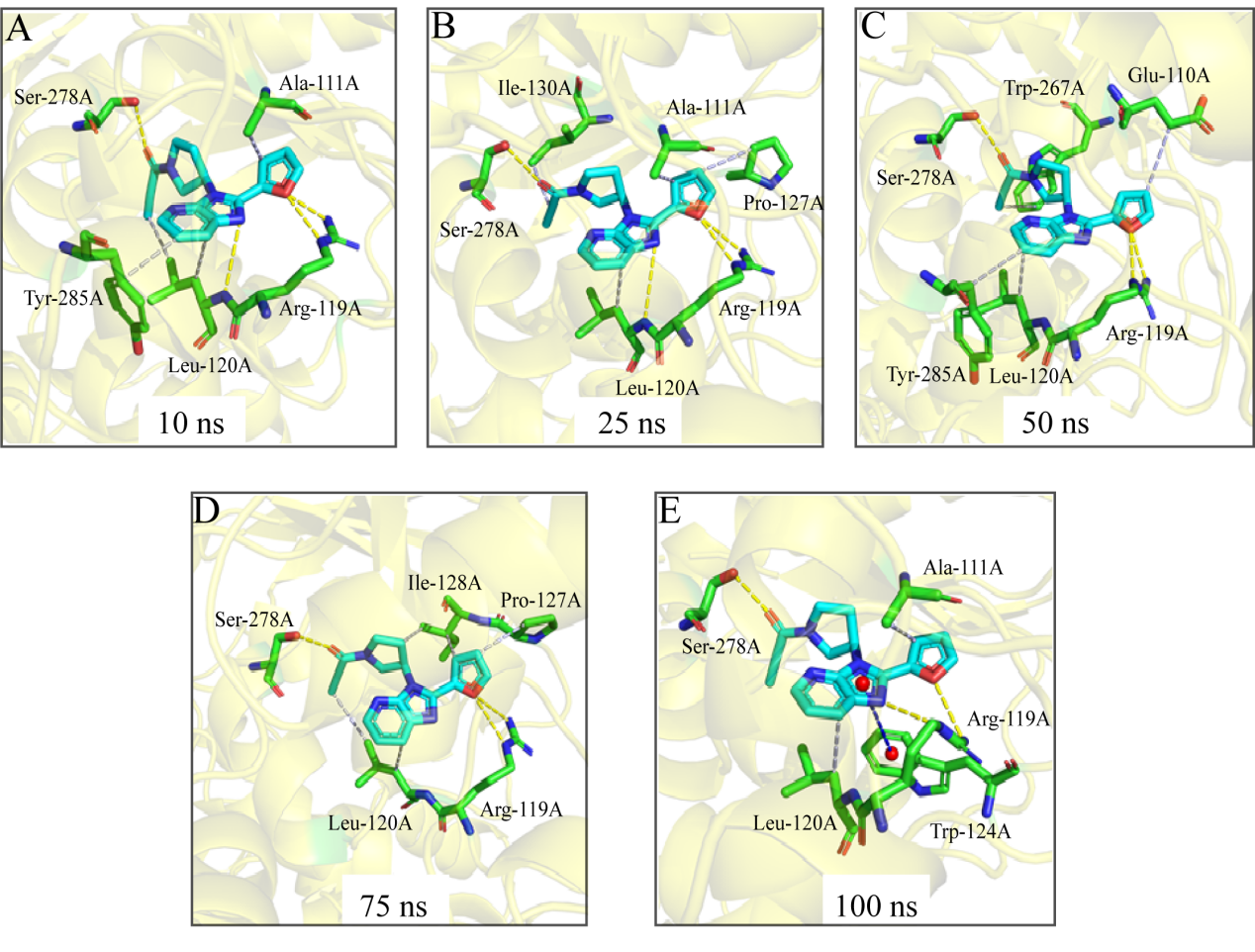


**Supplement Figure 5. Conformations of IDH1R132C-T001-0657 complex at different stages of MD simulation: (a) 10 ns, (b) 25 ns, (c) 50 ns, (d) 75 ns and (e) 100 ns.**

**
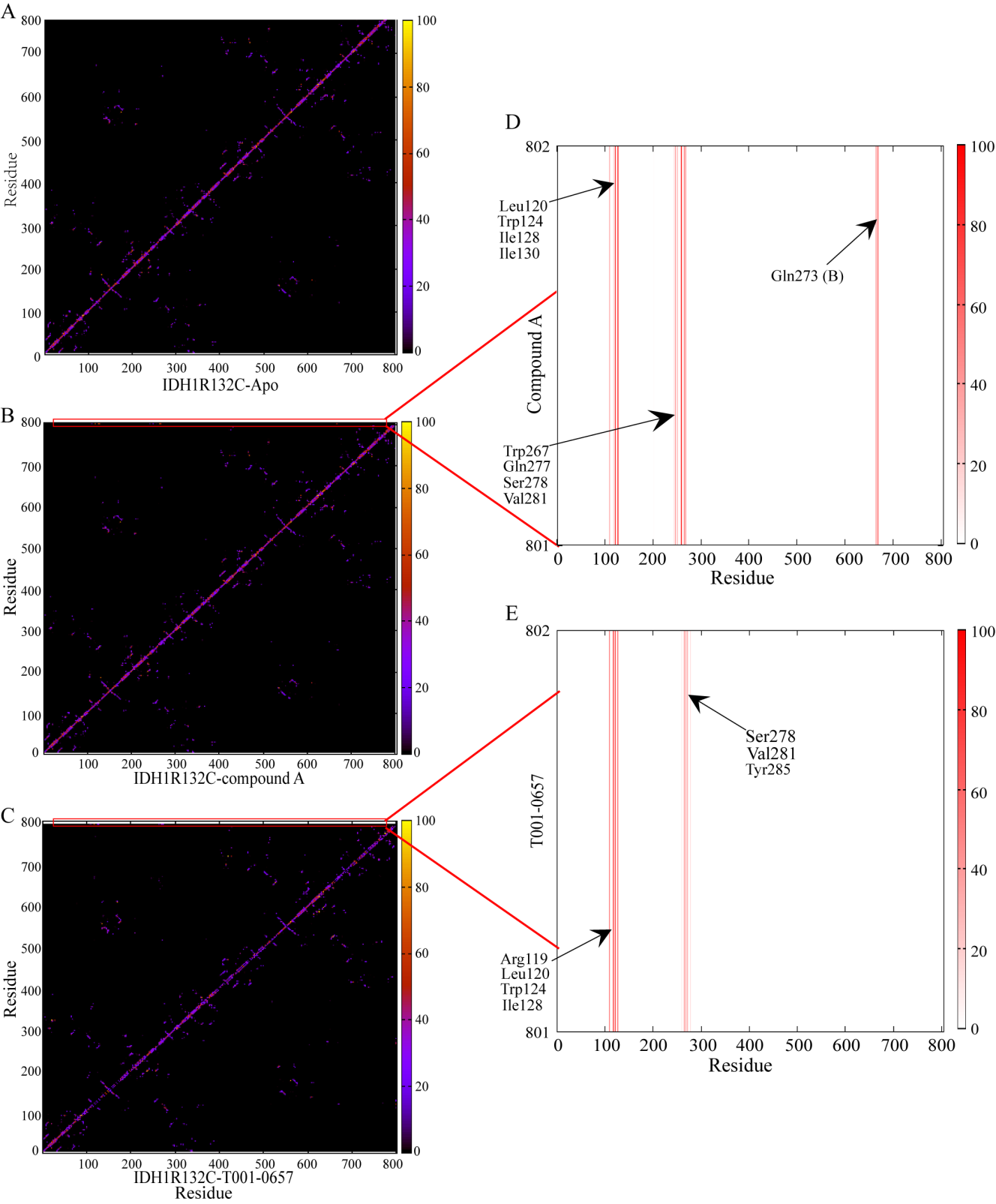
**

**Supplement Figure 6. (A)-(C).The contact maps for the IDH1R132C-Apo and IDH1R132C complexed compound A and T001-0657 in molecular dynamics simulations. (D) corresponding to the intermolecular contacts between IDH1R132C and compound A. (E) corresponding to the intermolecular contacts between IDH1R132C and T001-0657. The color bars indicate the relative contact strength.**

**
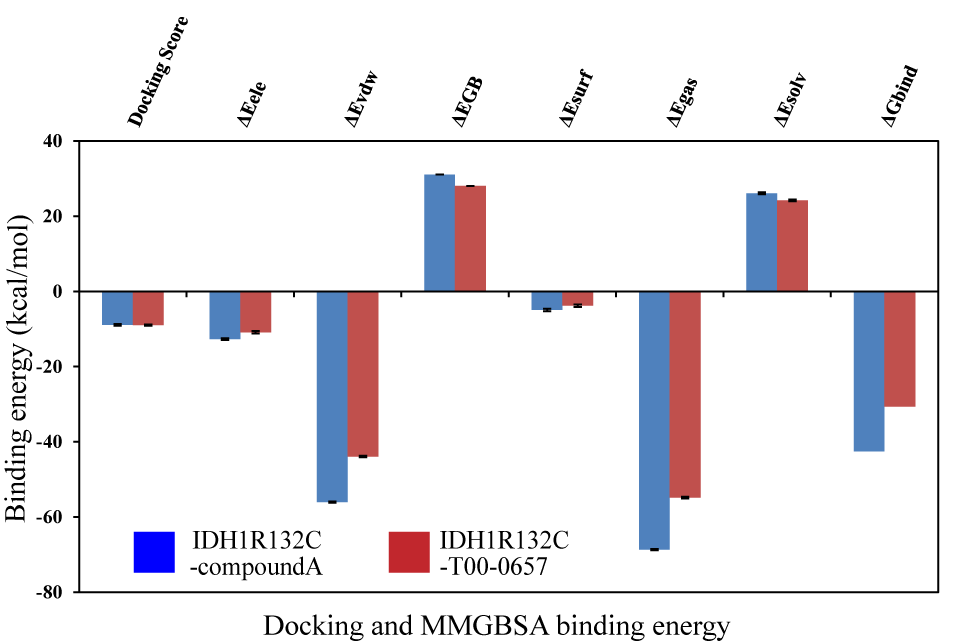
**

**Supplement Figure 7. Docking and MM-GBSA binding free energy (kcal/mol) of IDH1R132C-compoundA and IDH1R132C-T001-0657 complexs.**

Supplement Table 1. The virtual screening and molecular docking grid details (Docking Software, center points, grid size, and amino acid residues) of IDH1R132C.

| **Docking Software** | **Center Points** | | | **Grid size (Å)** | | | **Molecular grid amino acid residues** |
| --- | --- | --- | --- | --- | --- | --- | --- |
|  | **X** | **Y** | **Z** | **X** | **Y** | **Z** |  |
| Autodock Vina | 13.052 | -39.044 | 0.096 | 20 | 20 | 20 | ALA111,ARG119,LEU120,VAL121 |
|  |  |  |  |  |  |  | TRP124,ILE128,ILE130,CYS132 |
|  |  |  |  |  |  |  | ILE251,VAL255,ALA258,MET259 |
|  |  |  |  |  |  |  | TRP267,CYS269,GLN277,SER278 |
|  |  |  |  |  |  |  | VAL281,ALA282,TYR285,MET291 |
|  |  |  |  |  |  |  | GLN277(B),SER280(B),VAL281(B) |

Supplement Table 2. The physical characteristics and docking results of the 41 selected compounds

| **Compound** | **Docking Score(kcal/mol)** | **MW** | **HBD** | **HBA** | **ROB** | **HA** | **LogPo/w** | **TPSA** |
| --- | --- | --- | --- | --- | --- | --- | --- | --- |
| G384-0691 | -9.2 | 464.58 | 1 | 6 | 11 | 32 | 2.20 | 115.48 |
| G357-4730 | -9.1 | 475.49 | 1 | 7 | 10 | 35 | 4.01 | 112.14 |
| F588-0614 | -9.1 | 549.01 | 0 | 7 | 6 | 37 | 3.55 | 68.51 |
| F248-0087 | -8.9 | 388.40 | 4 | 6 | 8 | 27 | 1.18 | 181.29 |
| E947-0796 | -8.9 | 454.56 | 1 | 6 | 9 | 33 | 3.67 | 93.51 |
| E856-2279 | -8.8 | 482.53 | 1 | 5 | 8 | 36 | 3.50 | 85.69 |
| E760-3668 | -8.8 | 439.51 | 1 | 4 | 6 | 33 | 2.97 | 94.11 |
| E551-0249 | -8.7 | 538.64 | 1 | 8 | 10 | 37 | 2.91 | 152.47 |
| E239-0253 | -8.7 | 504.55 | 1 | 6 | 7 | 36 | 3.75 | 99.43 |
| D389-0647 | -8.6 | 487.55 | 0 | 5 | 6 | 36 | 3.98 | 91.36 |
| D302-0195 | -8.6 | 482.56 | 2 | 7 | 7 | 34 | 3.12 | 159.55 |
| D272-0767 | -8.6 | 409.87 | 3 | 3 | 7 | 29 | 3.36 | 79.14 |
| C885-4688 | -8.5 | 464.58 | 2 | 4 | 8 | 33 | 3.31 | 123.63 |
| C804-0735 | -8.5 | 431.48 | 2 | 4 | 8 | 32 | 3.18 | 95.58 |
| C804-0664 | -8.5 | 449.50 | 2 | 5 | 9 | 33 | 3.73 | 96.97 |
| C776-3686 | -8.4 | 473.61 | 1 | 3 | 6 | 35 | 3.69 | 84.46 |
| C561-1816 | -8.4 | 473.57 | 1 | 6 | 10 | 35 | 4.35 | 90.64 |
| C561-1792 | -8.4 | 463.57 | 1 | 6 | 10 | 34 | 4.42 | 90.64 |
| C561-1789 | -8.3 | 459.54 | 1 | 6 | 10 | 34 | 4.10 | 90.64 |
| C561-0552 | -8.3 | 472.58 | 1 | 5 | 10 | 35 | 4.52 | 77.75 |
| C519-2030 | -8.3 | 551.65 | 0 | 7 | 8 | 39 | 4.08 | 114.37 |
| C519-2005 | -8.3 | 566.67 | 0 | 7 | 9 | 40 | 4.24 | 117.61 |
| C406-0221 | -8.3 | 546.36 | 2 | 7 | 8 | 37 | 2.89 | 147.76 |
| C241-1890 | -8.3 | 496.54 | 1 | 5 | 8 | 36 | 3.02 | 138.27 |
| C241-1416 | -8.3 | 562.64 | 1 | 6 | 13 | 40 | 3.57 | 140.11 |
| C212-0123 | -8.3 | 574.09 | 2 | 5 | 12 | 40 | 4.58 | 125.75 |
| C190-0162 | -8.3 | 456.92 | 1 | 4 | 6 | 32 | 3.52 | 87.64 |
| C172-0032 | -8.2 | 494.99 | 1 | 3 | 5 | 34 | 3.57 | 106.65 |
| 8003-0276 | -7.9 | 429.34 | 1 | 8 | 4 | 32 | 2.01 | 127.28 |
| 5762-1874 | -7.9 | 371.77 | 2 | 4 | 5 | 26 | 2.00 | 101.73 |
| 5641-2197 | -7.9 | 411.45 | 2 | 5 | 8 | 30 | 2.58 | 110.96 |
| 4464-1033 | -7.9 | 582.69 | 4 | 7 | 20 | 42 | 3.56 | 165.92 |
| 4228-1435 | -7.8 | 384.41 | 2 | 6 | 7 | 27 | 2.31 | 129.31 |
| 4082-0684 | -7.8 | 375.78 | 1 | 4 | 2 | 26 | 2.76 | 71.41 |
| 3969-0703 | -7.8 | 584.64 | 1 | 8 | 11 | 42 | 4.37 | 144.66 |
| 3773-4485 | -7.8 | 552.67 | 4 | 6 | 12 | 40 | 2.80 | 128.69 |
| 3699-1233 | -7.8 | 523.94 | 3 | 6 | 5 | 36 | 2.59 | 161.03 |
| 3699-0083 | -7.8 | 552.33 | 3 | 7 | 5 | 36 | 2.31 | 146.01 |
| 2368-1306 | -7.8 | 431.42 | 1 | 5 | 4 | 32 | 2.71 | 117.23 |
| 2368-0992 | -7.8 | 413.47 | 1 | 3 | 3 | 31 | 3.35 | 71.41 |
| 1809-0342 | -7.8 | 449.89 | 2 | 6 | 8 | 32 | 2.99 | 130.11 |

Supplement Table3. The average RMSD, RMSF, RoG, SASA of compoundA and T001 of three replicas

| Complexes | Repeat times | RMSD | RMSF | RoG | SASA |
| --- | --- | --- | --- | --- | --- |
| compoundA | 1 | 3.66 | 6.63 | 28.84 | 79.93 |
|  | 2 | 3.54 |  | 28.81 | 72.05 |
|  | 3 | 3.92 |  | 29.26 | 85.49 |
| T001 | 1 | 2.96 | 5.96 | 28.89 | 69.58 |
|  | 2 | 3.55 |  | 29.12 | 92.37 |
|  | 3 | 2.79 |  | 28.75 | 50.27 |

Supplement Table4. The relationship between IC_50_ and ΔG

| compound | IC_50_ (μM) | ΔG_cal_ (kcal/mol) | ΔG_exp_ (kcal/mol) |
| --- | --- | --- | --- |
| compoundA | 0.13 | -9.43 | -42.58 |
| T001-0657 | 1.311 | -8.06 | -30.62 |

ΔG_cal_=−RT·pIC50, R= 8.314J·mol-1·K^-1^, T: temperature(K)

ΔG_exp_: Binding free energy contributions calculated by the MMGBSA method (kcal/mol).

Supplement Table5. The specific energy contribution values of the two compounds with IDH1R132C

|  | R132C-T001-0657 | R132C-compoundA |
| --- | --- | --- |
| GLU 110 | -0.15 | -0.47 |
| ALA 111 | -1.42 | -0.70 |
| LEU 120 | -1.53 | -1.17 |
| TRP 124 | -3.91 | -2.60 |
| PRO 127 | -0.20 | -1.50 |
| ILE 128 | -1.31 | -2.02 |
| ILE 130 | -1.21 | -1.69 |
| VAL 255 | -0.25 | -0.91 |
| MET 259 | -0.47 | -0.06 |
| TRP 267 | -0.42 | -2.35 |
| GLN 277 | -0.11 | -0.72 |
| SER 278 | -0.68 | -1.24 |
| ASP 279 | -0.13 | -0.58 |
| VAL 281 | -0.16 | -1.01 |
| ALA 282 | -0.92 | -0.32 |
| TYR 285 | -0.43 | 0.00 |
| MET 291 | -0.42 | -0.33 |
| LEU 120B | 0.03 | -1.12 |
| SER 280B | 0.00 | -0.62 |
| GLN 283B | -0.01 | -1.24 |
